# Does colchicine reduce mortality in patients with COVID-19 clinical syndrome? An umbrella review of published meta-analyses

**DOI:** 10.1016/j.heliyon.2023.e20155

**Published:** 2023-09-16

**Authors:** Mohammed I. Danjuma, Rana Sayed, Maryam Aboughalia, Aseel Hassona, Basant Selim Elsayed, Mohamed Elshafei, Abdelnaser Elzouki

**Affiliations:** a Department of Internal Medicine, Hamad Medical Corporation, Doha, Qatar; b Department of Internal Medicine, Weill Cornell College of Medicine, New York, Doha, Qatar; c NHS Grampian (Dr. Grays Hospital), Scotland, UK; d Department of Internal Medicine, Qatar University College of Medicine, Doha, Qatar; e Department of Pharmacy, Hamad Medical Corporation, Doha, Qatar

**Keywords:** COVID-19, Colchicine, Meta-analysis, Mortality, Umbrella

## Abstract

**Background:**

Despite significant improvements in both treatment and prevention strategies, as well as multiple commissioned reviews, there remains uncertainty regarding the survival benefit of repurposed drugs such as colchicine in patients with Coronavirus Disease 2019 (COVID-19) clinical syndrome.

**Methods:**

In this umbrella review, we carried out a comprehensive search of PubMed, EMBASE, Cochrane Database of Systematic Reviews, Science Citation Index, and the Database of Abstracts of Reviews of Effectiveness between January 1, 2020 and January 31, 2023 for systematic reviews and meta-analyses evaluating the mortality-reducing benefits of colchicine in patients with COVID-19. This was to ascertain the exact relationship between colchicine exposure and mortality outcomes in these cohorts of patients. We utilized A Measurement Tool to Assess systematic Reviews-2 (AMSTAR-2) to conduct an exhaustive methodological quality and risk of bias assessment of the included reviews.

**Results:**

We included eighteen meta-analyses (n = 199,932 participants) in this umbrella review. Colchicine exposure was associated with an overall reduction of about 32% in the risk of mortality (odds ratio 0.68, confidence interval [CI] 0.58–0.78; I2 = 94%, p = 0.001). Further examination of pooled estimates of mortality outcomes by the quality effects model (corrected for the methodological quality and risk of bias of the constituent reviews) reported similar point estimates (OR 0.73; CI 0.59 to 0.91; I2 = 94%).

**Conclusion:**

In a pooled umbrella evaluation of published meta-analyses of COVID-19 patient cohorts, exposure to colchicine was associated with a reduction in overall mortality. Although it remains uncertain if this effect could potentially be attenuated or augmented by COVID-19 vaccination.

## 1 Introduction Background

Coronavirus Disease 2019 (COVID-19) is a clinical syndromic illness caused by the SARS-COV-2 virus [1,2]. First diagnosed in 2019, it often starts as a prodromic respiratory illness, with a significant proportion of affected patients experiencing full recovery. Other affected patients however pursue a stormier course characterized endothelial dysfunction, cascading multi-organ involvement and death [2]. Successful immunization with a variety of approved Coronavirus Disease 2019 (COVID-19) vaccines has significantly contributed to containing both the spread and the adverse consequences of this nascent infection [3,4]. However, the exact role of clinical therapeutics in its management is still evolving [5]. Since the onset of the COVID-19 pandemic, repurposing of drugs with well-established marketing authorizations has formed part of therapeutic initiatives examined to determine their potential efficacy and safety in this cohort of patients [[6], [7], [8], [9], [10]]. One of these drugs is colchicine. Its potential utility in the treatment of COVID-19 patients has been examined in patient populations with different morbidity characteristics [[11], [12], [13], [14]]. Its role in COVID-19 infection has particularly been highlighted in the early and late phases of the disease. Survival outcomes in COVID-19 patients exposed to it were discordant, with some studies reporting a decreased proportion of mortality, and others showing a null effect on survival [[15], [16], [17], [18], [19], [20], [21], [22], [23], [24], [25]]. In order to conclusively determine the exact relationship between colchicine exposure and mortality in these cohorts, several meta-analyses have been commissioned and published, unfortunately with residual uncertainty [[15], [16], [17], [18], [19], [20], [21], [22], [23], [24], [25]]. The latter is due to a subset of these reviews reporting increased survival with colchicine, while others were associated with no effect on mortality. Yasmin et al. [18], for example, reported a significant reduction in mortality outcomes among patients exposed to colchicine compared to those on “usual” standard of care, while Chiu et al. [17], evaluating almost identical patient demographic populations, reported a *null* effect on mortality outcomes. The exact reason for these discordant outcomes remains unknown. Several factors have been suggested to account for this, including differences in the design of the constituent studies included in the secondary meta-analytical syntheses; differences in constituent patients’ populations; and lately COVID-19 vaccination status of patients included the reviewed studies. Whatever may be the exact reason for the discrepancy in these reported mortality outcomes, there is a residual need to explore further analytical pathways with the view to establishing the exact relationship between colchicine exposure and mortality outcomes in these cohorts. The recent flare of COVID-19 transmission in China and its resultant morbidity toll on the population sends a clear unequivocal reminder that there continue to be residual challenges with this virus both in terms of therapeutics and prevention. Therefore, to conclusively establish the exact relationship between colchicine exposure and mortality, we carried out comprehensive umbrella meta-analyses of published reviews since the onset of the pandemic.

## 2 Methods

**Fig. 1:**
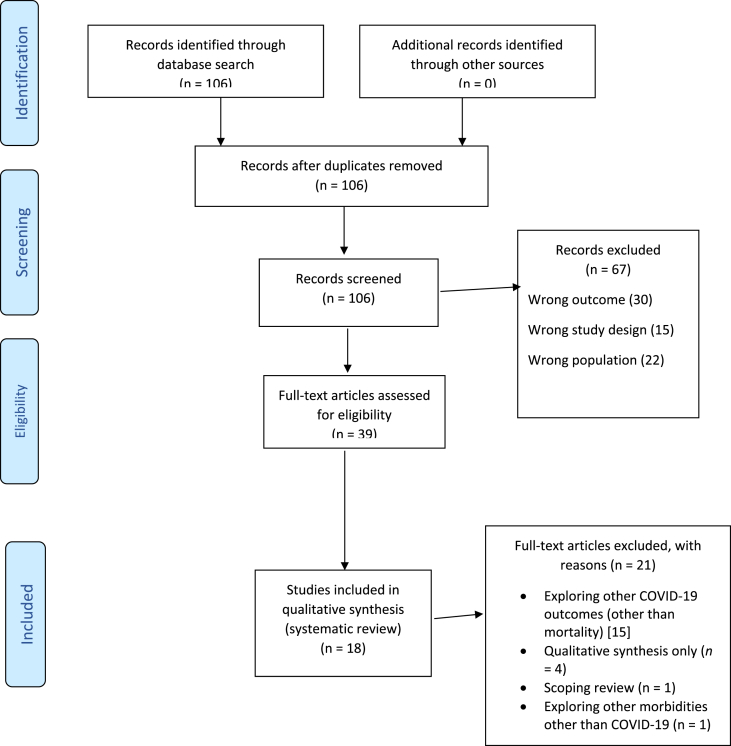
PRISMA Flow chart for study selection.

In conducting this review, we adhered to the Preferred Reporting Items for Systematic Reviews and Meta-Analyses (PRISMA) procedure [26] for the study selection as shown in Fig. 1. This umbrella review of published meta-analyses was registered in the PROSPERO database (**CRD42023397246**).

### 2.1 Data sources and literature search

We searched PubMed, EMBASE, Cochrane Database of Systematic Reviews, Science Citation Index, and database of abstracts of reviews of effectiveness between January 1, 2020 and January 31, 2023 for publications that satisfied prespecified inclusion criteria outlined in the review protocol. We used the following medical subject headings (MESH) terms to identify titles and abstracts for screening: (((colchicine) AND (COVID-19)) OR (Coronavirus 2 disease)) AND (mortality). We only included studies with meta-analytical designs exploring the efficacy and safety of colchicine in patients with COVID-19 infection.

### 2.2 Study selection

Following the initial literature search and removal of duplicate publications, we carried out a study eligibility assessment from the resulting abstracts. Two independent reviewers (RS and MA) assessed each study for inclusion in the review based on pre-specified inclusion criteria. In the unlikely event of disagreement between reviewers, this was usually resolved through consensus. Where consensus could not be reached, a third reviewer (MID) was called in to adjudicate. We drew up a final list of all included studies that met the following eligibility criteria: studies published in the English language between January 1, 2020, and January 31, 2023; patients over 18 years of age with COVID-19 infection; exposure to colchicine as part of study participant treatment regimen; reviews providing pooled estimates of mortality following colchicine exposure. We excluded all studies that failed to meet the inclusion criteria.

### 2.3 Data Extraction and study quality assessment

We extracted the following variables from each included study: last name of the first author and the year of publication; study centre/location; the number of COVID-19 patients; odds ratio of mortality outcomes reported by the meta-analysis and the model (random or fixed effects) under consideration. Where alternative effect sizes were given (such as risk ratios or relative risks), these were converted to odds ratios before pooled analyses.

We conducted a quality and risk of bias assessment using A Measurement Tool to Assess Systematic Reviews-2 (AMSTAR-2) tool [27]. An exhaustive description of this tool is given elsewhere [27]. Each included meta-analytical review was examined against sixteen quality safeguards to assess their risk of bias and methodological quality. Two independent reviewers (BE and AH) utilized this tool to conduct screening for the methodological quality of the included studies. In the unlikely event of disagreement between them, this was resolved by consensus or by the third reviewer (MID). The resulting scores from the quality assessment by AMSTAR-2 were subsequently rescaled into ranks between 0 and 1. The latter represents studies with the least risk of bias. These ranks were incorporated into the quality effects model to adjust estimates of mortality following colchicine exposure.

### 2.4 Statistical analysis

Descriptive statistics such as median and interquartile range were estimated for age and sample size, whilst categorical data was reported as their respective frequencies and percentages. For studies included in the umbrella review, before we quantify the pooled estimates, we initially converted all reported effect sizes to odd ratios (where alternative effect sizes were reported). We then computed the pooled estimates of mortality from the included meta-analyses. We subsequently carried out umbrella meta-analyses using the fixed, random (DerSimonian-Laird), and quality effect models to ascertain the exact point estimate of mortality in COVID-19 patients exposed to colchicine. The quality effects (QE) model reduces estimator variances by redistribution of study weights through prior rescaling of quality ranks (from 0 to 1). We assessed heterogeneity between studies with *I*^*2*^ statistics. We will assume the *I*^*2*^ thresholds of 25%, 50%, and 75% to represent low, moderate, and high between-study variances, respectively. Results were presented as forest plots with odds ratio (OR) estimates for each of the included reviews and overall final pooled OR. We visualized small study effect and publication bias with a funnel and Doi plots and inference was made using Egger's tests. All analyses were conducted with MetaXL, version 5 (Epigear International, Sunrise Beach, QLD, Australia; www.epigear.com)

## 3 Results

### 3.1 Study selection

Following an exhaustive search of relevant databases (PubMed, Embase, and Scopus), we retrieved 106 studies with no duplicates. Following full-text screening, 16 articles (*n* = 194410.0 participants) (17–18, 20–23, 28–37, out of 39 met prespecified inclusion criteria and were included in the review. Three studies [[28], [29], [30]] were excluded because of their qualitative design with no [31]pooled estimates of mortality outcomes. Zhang et al. [31], and Cheng et al. [24] were excluded based on their Bayesian network meta-analytical design. Although Han et al. [32] did explore all range of therapeutic options in patients with severe acute respiratory syndrome (SARS), Middle East respiratory Syndrome (MERS), and COVID-19 since the onset of the pandemic, the review failed to provide a numerical point estimate of either efficacy or safety of colchicine exposure from their study. The number of studies evaluated by the 18 metanalyses ranged between three [33] to seventeen [34]. Fig. 1 shows the PRISMA flow diagram of the studies included in the umbrella synthesis.

### 3.2 Patient population

Table 1 shows the socio-demographic characteristics of the study population. The mean age of the reviewed population was 60.1 ± 3.6 years, 59.4% of which were males. The duration of colchicine exposure in the included meta-analyses was 28 days (interquartile range [IQR] 6, 99).

**Table 1:** characteristics of meta-analytical reviews reporting on mortality outcomes in Covid-19 patients exposed to Colchicine as part of their standard of care.

Study	Age (years)	Proportion of male gender (%)	Country of publication	Total sample size	Percentage of population vaccinated against Covid-19	Number of studies reviewed	Design of the included studies	Effect on mortality
Bitar 2022 [29]	58.4	63.3	Malaysia	18956	Not provided	17	All RCT's	Not reduced
Chiu 2021 [14]	62.1	58.1	USA	16248	Not provided	8	All RCT's	Reduced
De-Miguel-Balsa 2021 [21]			Spain	17377	Not provided	11	5 RCTs + 6 observational	Not reduced
Yasmin 2022 [15]	58.7	48.6	USA	16048	Not provided	5	All RCTs	Not reduced
Golpour 2021 [17]			Iran	5901	Not provided	10	All RCT's	Reduced
Hariyanto 2021 [30]	63.4	32.3	Indonesia	5778	Not provided	8	3 RCTs + 6 observational	Reduced
Kow 2021 [26]	56.3		Malaysia	17976	Not provided	10	All RCT's	Not reduced
Lan 2022 [27]			China	16024	Not provided	7	All RCT's	Not reduced
Lien 2021[35]]	60.8	62.9	Taiwan	17205	Not provided	11	4 RCTs and 7 observational	Reduced
Nawangsih 2021 [[36]]	59.4	43.6	Indonesia	5530	Not provided	8	3 RCTs + 6 observational	Reduced
Romeo 2022 [18]	57.4	58.3	Argentina	19271	Not provided	11	All RCT's	Not reduced
Salah 2021 [[37]]			US	5259	Not provided	7	All RCT's	Reduced
Toro-Huamanchumo 2022 [34]	63.0	61.6	Peru	13478	Not provided	9	5 RCTs and 4 observational	Not reduced
Vrachatis 2021 [19]	51.4	56.9	Greece	881	Not provided	6	–	Reduced
Zein 2022 [13]	61.4	60.4	Indonesia	6953	Not provided	12	4 RCTs and 8 observational	Reduced
Mikolajewska [[38]]	64.0	62.8	Germany	16013	Not provided	4	All RCTs	Not reduced
Crichton 2021 [28]			UK		Not provided	3	All RCTs	Not reduced
Elsafei 2021 [12]	61.3	59.8	Qatar		Not provided	9	3 RCTs, one quasi-experimental, and 5 observations	Reduced

### 3.3 Effect of colchicine on mortality

**Fig. 2a:**
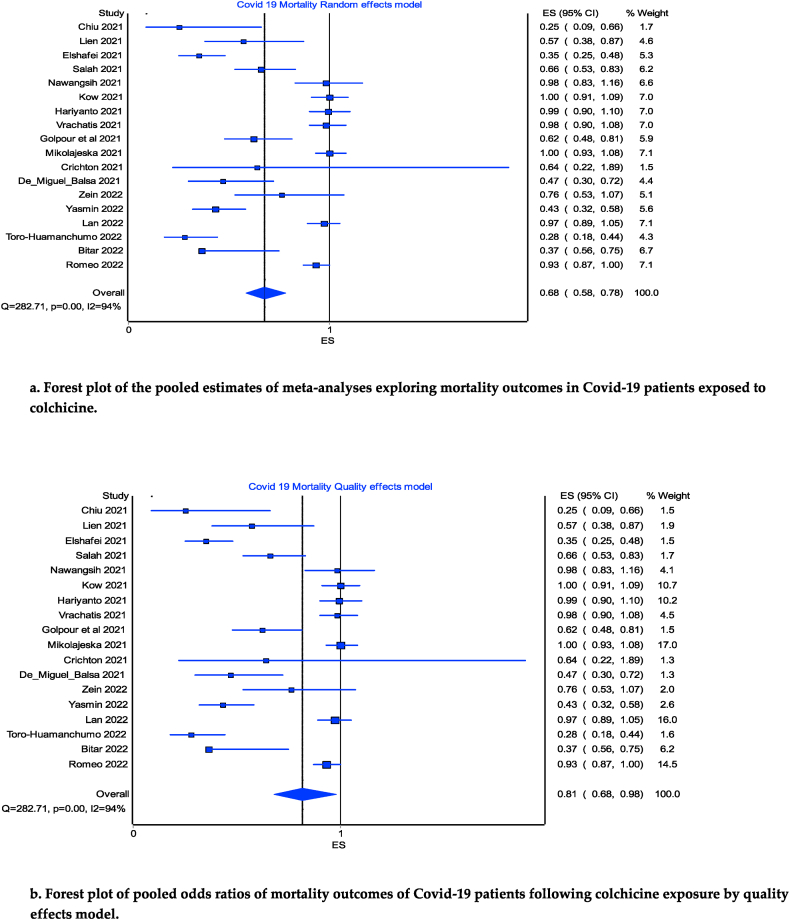
Forest plot of the pooled estimates of meta-analyses exploring mortality outcomes in Covid-19 patients exposed to colchicine. Fig. 2b. Forest plot of pooled odds ratios of mortality outcomes of Covid-19 patients following colchicine exposure by quality effects model.

**Fig. 3:**
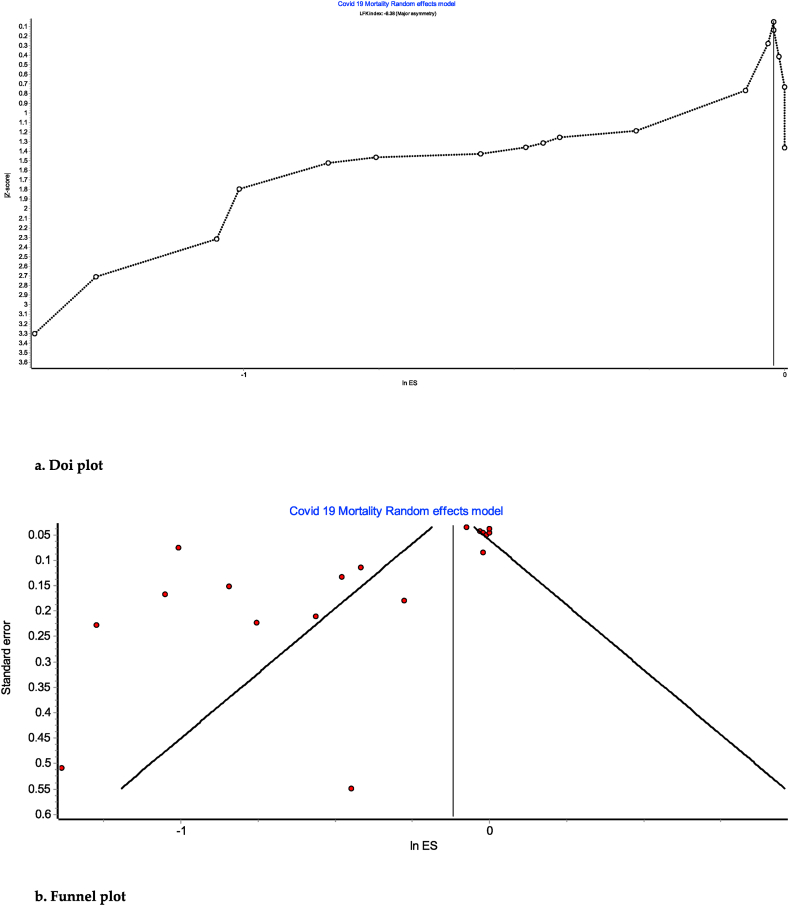
a. Doi plot Fig. 3b. Funnel plot.

In a pooled analysis by random effects model, exposure to colchicine was associated with about a 35% overall reduction in mortality (Odds ratio 0.68, confidence interval [CI] 0.58–0.78; *I*^*2*^ = 94%, p = 0.001). Further examination of pooled estimates of mortality outcomes by the quality effects model (corrected for the methodological quality and risk of bias of the constituent reviews) reported estimates within the same ballpark (OR 0.73; CI 0.59 to 0.91; *I*^*2*^ = 94%) Fig. 2a and b. Crichton et al.’s synthesis is particularly instructive as it formed the therapeutic basis for the European Respiratory society's living guideline recommendation for the treatment of COVID-19 patients [33]. That this guidance advised against the utility of colchicine for the treatment of these cohorts of patients owing to its distinct lack of survival benefit from their synthesis (OR 0.64 [0.22–1.89], *I*^*2*^ = 34%, P = 0.34). Vrachatis et al. review is the first investigation of the pooled estimate of colchicine mortality benefit in patients with COVID-19 in January 2021 [22]. This synthesis is limited to the review of just five studies due mainly to the paucity of published reports in the initial phase of the pandemic. It only reported a signal of colchicine administration added to the “usual standard of care” (OR 1.0 [CI 0.9–1.1], *I*^*2*^ = 0.00%, P = 0.00). About 12 months after this latter report, Kow et al. [39], and Chiu et al. [17] both carried out and published updated meta-analyses of additional observational studies and RCTs that have accrued on COVID-19 patients exposed to colchicine. In a pooled analyses of ten RCTs (n = ), Kow et al. found no significant difference in mortality outcomes between COVID-19 patients on colchicine *vs*. those on what was still evolving as “usual standard of care then (pooled OR = 0.76; 95% CI: 0.53–1.07, *I*^*2*^ = 26%, P = 0.21) [39]. Conversely, Chiu et al.’s., examination of eight studies found a lower risk of mortality among patients who received colchicine compared to controls (OR of 0.22 [95% CI: 0.09, 0.57]) [17]. It is noteworthy that the pivotal RECOVERY-2 trial [40] was amongst the studies reviewed in this meta-analysis. S2 depicts the result of sensitivity analyses following the sequential exclusion of various meta-analytical reviews (see Fig. 3).

### 3.4 A cumulative review of the evidence by year of publication

We additionally carried out an iterative synthesis of the time of publication of the meta-analyses with the view to ascertain exactly when the evidence for reducing mortality stabilizes.Figure S1shows the forest plot of sequential weighted effects of meta-analyses in succeeding years since the onset of the pandemic. The evidential threshold for positive colchicine effect on mortality was probably “mature” by the end of 2021.

**The influence of** COVID-19 **Vaccination**.

None of the primary studies included the 18 meta-analyses we examined in this umbrella review reported on populations who have received COVID-19 vaccination. It is instructive that this included Yasmin et al. [18], Bitar et al. [34], Romeo et al. [21], and Toro-Huamanchumo et al. [41] all of which were published in 2022 when COVID-19 vaccination had assumed the status of “standard of care” by most international treatment guidelines [42].

### 3.5 Sub-group analyses

When we explored the effect of external variables such as study population on the overall heterogeneity by categorizing studies with >10000 patients as “large” studies, with those with lesser patient thresholds as “small”. We found no difference in the relative proportion of heterogeneity between the two categories (96% vs. 87% for “large” and “small” studies respectively). Similarly, we found similar heterogeneity estimates amongst studies with a predominant population above and below 60 years of age (90% vs, 97% respectively) S3 and S4.

### 3.6 Assessment of heterogeneity

We found significant heterogeneity amongst the studies included in this umbrella review (pooled *I*^*2*^ of 94%), with major asymmetry apparent by both funnel and Doi plots (Luis Furuya-kanamori [LFK] index 6.28). This is probably due to the heterogeneity of the patient population and differences in the design of studies included in the meta-analyses reviewed by this umbrella review. See Fig. 3a and b.

### 3.7 Small study effects and publication bias

Quality and methodological assessment of the included studies demonstrated that a significant Proportion of them included key components essential for robust PICO assessment of the review, explanation of inclusion criteria, justification why some studies were excluded, as well as the utility of a satisfactory well-validated quality assessment tool (AMSTAR 2) [27,43]. The median quality scores (from the ranked AMSTAR-2 scores) of the included meta-analyses examining mortality outcomes in these cohorts of patients was 5.5 (IQR 2.75, 7.25). For an exhaustive description of the included studies See supplementary material S1. We rate the methodological quality of included meta-analyses as low, moderate, and high. The principal source of bias in the reviewed studies was lack of clarity regarding reason (s) for inclusion of studies with different designs into their meta-analysis.

## 4 Discussion

In this first umbrella review of the effects of colchicine administration on mortality outcomes in patients with COVID-19, we found patients exposed to colchicine had about 32% reduced risk of mortality compared to those stabilized on the usual standard of care only. This estimate is against a background of heterogenous pooled meta-analyses with diverse methodological and socio-demographic foundation (*I*^*2*^ of 94%). With the successful identification and confirmation of the mortality-reducing properties of COVID-19 vaccination (amongst other measures), the standard of acceptability of new and repurposed drugs in the therapeutics of COVID-19 clinical syndrome has changed [42]. Reducing morbidities such as duration of illness, number of hospitalizations, and other morbidity-related sequalae may not be as wholly satisfactory as they were in the early phase of the pandemic. During this time emphasis was rightly placed on identifying putative agents (any putative agent) that could slow both the spread of the virus as well as management of patients with overt clinical syndrome. In addition to novel therapeutic agents, repurposed drugs with well-established marketing authorizations were also explored with the view to ascertaining their efficacy and safety in patients with COVID-19 [[6], [7], [8], [9], [10], [11], [12], [13], [14]]. Colchicine has been the subject of recent concerns. The exact mechanisms underlying colchicine effect on COVID-19 viral dynamics is still uncertain but is suggested to include its non-specific inhibition of the NACHT, leucine-rich repeat, and pyrin domain-containing protein 3 (NLRP3) inflammasome [44]. The latter has been proposed to mediate the downstream release of interleukin-6 (IL-6), a key cytokine in COVID-19 pathogenicity [44]. Colchicine in common with other drugs such as Lopinavir, Favipiravir, systemic steroids, tocilizumab [10,[45], [46], [47]], etc. was extensively tested with conflicting results in patient populations with COVID-19 clinical syndrome. Unfortunately, as was evident in the initial phase of the pandemic, studies examining the efficacy and safety of colchicine in these patient cohorts reported different outcomes, especially with regards to its effect on reducing mortality. Subsequent meta-analyses commissioned to examine and resolve any lingering uncertainty regarding the exact relationship between colchicine exposure and mortality outcome, unfortunately, reported largely discordant outcomes themselves. For example, while review by Elshafei et al. [15], showed a survival benefit of colchicine, others such as those by Lan et al. [48], and Yasmin et al. [18] reported a *null* effect of colchicine exposure. Having a combined qualitative synthesis with pooled estimates of the various meta-analyses (in form of an umbrella review) that have examined mortality outcomes *vis-à-vis* colchicine exposure in the COVID-19 patient population was therefore long overdue.

COVID-19 immunization in common with other measures such as social distancing and face masks represents the combined package that significantly slowed the transmission of the virus and reduce nearly all phenotypes of COVID-19 morbidity and mortality [[1], [2], [3]]. The fact that none of published meta-analytical syntheses carried out a themed assessment of the effect of Covid-19 vaccination on colchicine mortality outcomes meant that uncertainty regarding this still subsist. In a recent mechanistic commentary on the possible impact of colchicine on Adenovirus vector based COVID-19 vaccines, Lin et al. [49] suggested that colchicine could potentially hinder the delivery of Adenovirus genome (of the ChAdOx1 nCoV-19 vaccine) to vaccinated host cell's nucleus; the latter is a requisite step in Adenoviral genome transition post vaccination. Suggested mechanism for this has been postulated to include colchicine alteration with the microtubule movement through suppression of microtubule dynamics at lower concentrations and induction of depolymerization of microtubular architecture at higher concentrations [49].

The disparities in mortality outcomes evident in the point estimates of the various meta-analyses could be attributable to differences in design of the constituent studies included in their synthesis. In the early phase of the pandemic where clinical emphasis was solely placed on identifying effective and safe treatment of the clinical syndrome, a plurality of studies reported during this period had undeniably poor design *vis-à-vis* the clinical questions they attempted to investigate. Additionally, often measured outcomes from these reports were unavoidably confounded by concomitant and sometimes necessary administration of other putative agents. The latter creates a milieu of clinical interaction potentially confounding measured outcomes (such as mortality) that is difficult to disentangle or attribute to a particular drug. Additionally, efficacy and safety assessment of novel and repurposed therapeutics in the early phase of the pandemic suffered from the phenomenon of “confounding by indication”. As what was perceived as “clinical improvement” and or “deterioration” following drug administration may in fact be the natural history of a new disease process (COVID-19) that wasn't fully understood then. Vrachatis et al.’s [22]. meta-analytical synthesis been the first to explore uncertainty surrounding colchicine's role in reducing COVID-19 mortality outcomes was seriously confounded by the exclusive observational non-randomized design of its constituent studies. With subsequent reviews, there was more certainty regarding the pooled estimates of mortality, principally due to rising numbers of published RCTs [50](33) [18].

## 5 Summary of findings

The result of this umbrella review read together with recent RCTs exploring outcomes in COVID-19 patients [such as Perricone et al. [51]] will suggest that colchicine does possess mortality reducing properties in COVID-19 patients exposed to it, but how this effect is enhanced or attenuated by concomitantly tested therapeutics remains unknown. In the light of the fact that a significant proportion of the patient population included in this review were unvaccinated with COVID-19 vaccine, it remains uncertain what role this may have played either augmenting or attenuating its effect on mortality.

### 5.1 Strength and limitations

This umbrella review represents the first and only comprehensive examination of published meta-analyses exploring mortality outcomes in patients with COVID-19, and thus has the potential to inform practice going forward. The relative spread of the constituent reviews throughout the critical years of the pandemic (July 2020 to December 2022) meant that the studies it examined represents the “core” of COVID-19 morbidities and the uncertainty surrounding how best to manage them.

This review is limited by confounding factors native to the constituent meta-analyses and the primary studies they reviewed. Additionally, consistent with known flaws of our study design, the validity of umbrella reviews such as ours is pretty much “held hostage” by the methodological quality of the constituent meta-analyses we reviewed. Our determination of pooled estimates of odd ratios using the quality effects model was aimed at mitigating some of these biases. Furthermore, the fact that the population reviewed by this umbrella synthesis was unvaccinated against COVID-19, suggests caution in the generalizability of our findings to the larger COVID-19 population.

## 6 Conclusion

In a pooled umbrella evaluation of published meta-analyses of COVID-19 patient cohorts, exposure to colchicine was associated with a reduction in overall mortality. Although it remains uncertain if this effect could potentially be attenuated or augmented by COVID-19 vaccination.

## Funding

This review has no funding affiliation to declare. Open Access funding provided by the Qatar National Library

## Ethics

Not applicable.

## Availability of data

Data generated and utilized for analyses of results presented in this manuscript are available from the corresponding author on reasonable requests.

## Author contribution statement

All authors listed have significantly contributed to the development and the writing of this article.

## Data availability statement

Data will be made available on request.

## Additional information

No additional information is available for this paper.

## Declaration of competing interest

The authors declare that they have no known competing financial interests or personal relationships that could have appeared to influence the work reported in this paper.
