# Supplementary material for: Does colchicine reduce mortality in patients with COVID-19 clinical syndrome? An umbrella review of published meta-analyses
Source: Heliyon. 2023 Sep 16;9(10):e20155. doi: 10.1016/j.heliyon.2023.e20155 (PMC10520783; doi:10.1016/j.heliyon.2023.e20155)
Supplement: Multimedia component 1 [file mmc1.docx]

**Supplementary files**

**S1.** Cumulative meta-analysis of year of publication of meta-analyses evaluating mortality outcomes in COVID-19 patients on colchicine as part of their standard of clinical care.

**S2 Result of sensitivity analyses of the reviewed meta-analytical synthesis.**

| Excluded study | Pooled Odds ratios | LCI 95% | HCI 95% | Cochran Q | p | *I ^2^* | I 2 LCI 95% | I 2 HCI 95% |
| --- | --- | --- | --- | --- | --- | --- | --- | --- |
| Chiu 2021 | 0.69 | 0.59 | 0.80 | 276.47 | 0.00 | 94.21 | 92.10 | 95.76 |
| Lien 2021 | 0.68 | 0.59 | 0.79 | 278.25 | 0.00 | 94.25 | 92.16 | 95.78 |
| Elshafei 2021 | 0.70 | 0.61 | 0.81 | 251.00 | 0.00 | 93.63 | 91.22 | 95.37 |
| Salah 2021 | 0.68 | 0.58 | 0.79 | 275.77 | 0.00 | 94.20 | 92.08 | 95.75 |
| Nawangsih 2021 | 0.66 | 0.56 | 0.77 | 281.38 | 0.00 | 94.31 | 92.25 | 95.83 |
| Kow 2021 | 0.65 | 0.56 | 0.76 | 275.40 | 0.00 | 94.19 | 92.07 | 95.75 |
| Hariyanto 2021 | 0.65 | 0.56 | 0.77 | 277.89 | 0.00 | 94.24 | 92.14 | 95.78 |
| Vrachatis 2021 | 0.65 | 0.56 | 0.77 | 277.82 | 0.00 | 94.24 | 92.14 | 95.78 |
| Golpour et al 2021 | 0.68 | 0.58 | 0.79 | 275.29 | 0.00 | 94.19 | 92.06 | 95.74 |
| Mikolajeska 2021 | 0.65 | 0.55 | 0.77 | 271.36 | 0.00 | 94.10 | 91.94 | 95.69 |
| Crichton 2021 | 0.68 | 0.58 | 0.78 | 282.35 | 0.00 | 94.33 | 92.28 | 95.84 |
| De_Miguel_Balsa 2021 | 0.69 | 0.59 | 0.80 | 274.51 | 0.00 | 94.17 | 92.04 | 95.73 |
| Zein 2022 | 0.67 | 0.58 | 0.78 | 281.93 | 0.00 | 94.32 | 92.27 | 95.83 |
| Yasmin 2022 | 0.70 | 0.60 | 0.81 | 259.49 | 0.00 | 93.83 | 91.53 | 95.51 |
| Lan 2022 | 0.65 | 0.55 | 0.77 | 277.82 | 0.00 | 94.24 | 92.14 | 95.78 |
| Toro-Huamanchumo 2022 | 0.71 | 0.61 | 0.81 | 256.88 | 0.00 | 93.77 | 91.44 | 95.47 |
| Bitar 2022 | 0.74 | 0.66 | 0.83 | 139.56 | 0.00 | 88.54 | 83.22 | 92.17 |
| Romeo 2022 | 0.65 | 0.55 | 0.77 | 280.77 | 0.00 | 94.30 | 92.23 | 95.82 |

**S3: A Forest of exploratory analyses of the effect of Age on the heterogeneity estimates of the review.**

**S4: A Forest plot depicting the null effect of categorized age on the heterogeneity estimates of the review.**
